# Supplementary material for: Exploring patient-, provider-, and health facility-level determinants of blood pressure among patients with hypertension: A multicenter study in Ghana
Source: PLOS Glob Public Health. 2024 Jul 15;4(7):e0002121. doi: 10.1371/journal.pgph.0002121 (PMC11249229; doi:10.1371/journal.pgph.0002121)
Supplement: S2 Table — (PDF) [file pgph.0002121.s003.pdf]

Hill-Bone HBP Compliance to High Blood Pressure Therapy Scale (HB-HBP)

No.	Item	Response:<br>1. All of the Time<br>2. Most of the Time<br>3. Some of the Time<br>4. None of the Time
1	How often do you forget to take your **high blood pressure** medicine?	
2	How often do you decide NOT to take your **high blood pressure** medicine?	
3	How often do you eat salty food?	
4	How often do you shake salt on your food before you eat it?	
5	How often do you eat fast food?	
6	How often do you make the next appointment before you leave the doctor's office?*	
7	How often do you miss scheduled appointments?	
8	How often do you forget to get prescriptions filled?	
9	How often do you run out of **high blood pressure** pills?	
10	How often do you skip your **high blood pressure** medicine before you go to the doctor?	
11	How often do you miss taking your **high blood pressure** pills when you feel better?	
12	How often do you miss taking your **high blood pressure** pills when you feel sick?	
13	How often do you take someone else's **high blood pressure** pills?	
14	How often do you miss taking your **high blood pressure** pills when you are careless?	

* Reverse coding

Note:
Scale and subscale scores are calculated by summing individual items.
Reducing sodium intake subscale: Items 3,4,5
Appointment keeping subscale: Items 6,7
Medication taking subscale: Items 1, 2, 8,9,10,11,12,13,14
