# Supplementary material for: Exploring patient-, provider-, and health facility-level determinants of blood pressure among patients with hypertension: A multicenter study in Ghana
Source: PLOS Glob Public Health. 2024 Jul 15;4(7):e0002121. doi: 10.1371/journal.pgph.0002121 (PMC11249229; doi:10.1371/journal.pgph.0002121)
Supplement: S3 Table — (DOCX) [file pgph.0002121.s004.docx]

Healthcare Providers’ Knowledge and adherence to hypertension guidelines

The ADHINCRA Study

*Addressing Hypertension Control in Africa*

| **SECTION 1: DEMOGRAPHIC INFORMATION** | | |  |
| --- | --- | --- | --- |
| **Question** | | **Response** |  |
| A1 | Which health facility do your currently work in? | ___________________________ |  |
| A2 | Are you a: (PLEASE CHECK ONE) | 1. Physician/doctor 2. Registered Nurse 3. Community Health Nurse 4. Community Health Officer 5. Community Health Worker 6. Other _____________ |  |
| A3 | Gender (PLEASE CHECK ONE) | 1. Male 2. Female 3. Other __________ |  |
| A4 | How old are you? | _________ (years) |  |
| A5 | What is the **highest level of education** you have completed? (PLEASE CHECK ONE BOX) | 1. Junior secondary school completed 2. Senior secondary school completed 3. College/University completed 4. Post graduate degree 5. Refused 6. I don’t know |  |
| A6 | What is your **marital status?** (PLEASE CHECK ONE BOX) | 1. Never married 2. Currently married/Cohabitating 3. Separated 4. Divorced 5. Widowed 6. Refused |  |
|  | A7 | How many years have you spent working in Nursing/Medicine/clinical care? | ________ year(s)/______months |
|  | In which department do you currently work? |  |  |
| A8 | How many years have you spent treating patients with hypertension? | __________years/__________months |  |
| A9a | Have you taken continuing courses/training regarding hypertension in the previous 2 years? (PLEASE CHECK ONE BOX) | 1. Yes 2. No |  |
| A9b | If you answered **yes** to question A9a, what type of course/training did you receive? | _______________________________ |  |
| A9c | If you answered yes to question A9a, what was the duration of your course/training | _______________________________ |  |

CORRECT ANSWERS ARE IN RED

| **SECTION 2: HYPERTENSION KNOWLEDGE** | | |
| --- | --- | --- |
| **Question** | | **Response** |
| B1 | Which of the following statements regarding patient preparation for blood pressure measurement is correct?  (PLEASE CHECK ONE BOX) | 1. The patient should refrain from taking blood pressure medication on the day of their office visit. 2. **The patient should rest for at least 5 minutes prior to blood pressure measurement.** 3. Consumption of caffeine or tobacco just prior to the office visit will not affect blood pressure. 4. Patients should refrain from emptying their bladder prior to blood pressure measurement, in case a urine specimen is needed |
| B2 | Which of the following statements regarding patient positioning for blood pressure measurement is correct?  (PLEASE CHECK ONE BOX) | 1. The patient’s legs should hang freely from the edge of the examining table during blood pressure measurement. 2. The patient should hold his/her arm away from the body during blood pressure measurement. 3. The cuffed arm should be above the level of the heart during blood pressure measurement. 4. **The patient’s back should be supported during blood pressure measurement.** |
| B3 | Which of the following statements about placement of the blood pressure cuff is correct?  (PLEASE CHECK ONE BOX) | 1. Placing the cuff of an automatic (oscillometric) blood pressure device over a thin shirt sleeve is acceptable. 2. The blood pressure cuff should be centered over the radial artery. 3. It is acceptable to use a cuff that is too large for the patient’s arm, but not one that is too small. 4. **Placement of the cuff on the forearm is recommended in obese patients**. |
| B4 | Which of the following statements about blood pressure measurement is correct?  (PLEASE CHECK ONE BOX) | 1. An irregular heartbeat has no effect on automatic (oscillometric) blood pressure measurement. 2. Manually deflating the cuff too rapidly (more than 2 mmHg per second) will result in blood pressure readings that are too low. 3. **When taking multiple blood pressure measurements during a single office visit, it is important to wait at least 5 minutes between blood pressure measurements.** 4. When taking multiple blood pressure measurements during a single office visit, it is unusual for the readings to differ significantly from one another. |
| B5 | Which of the following statements regarding blood pressure measuring equipment is correct? | 1. Automatic (oscillometric) blood pressure devices should be calibrated daily. 2. **Blood pressure cuffs should be inspected regularly for leaks.** 3. Mercury sphygmomanometers have been removed from most clinics because of concerns about their accuracy. 4. Most home blood pressure monitors require use of a stethoscope by the patient. |
| B6 | If someone’s blood pressure is 115/75, it is | (0) High _____ (1) Low_____  **(2) Normal ______** (3) I don’t know ______ |
| B7 | If someone’s blood pressure is 160/100, it is | **(0) High _____** (1) Low_____  (2) Normal ______ (3) I don’t know ______ |
| B8 | Once someone has high blood pressure it usually lasts for | (0) A few years ________  (1) 5-10 years _________  **(2) The rest of their life ________**  (3) I don’t know ________ |
| B9 | People with high blood pressure should take their medicine | **(0) Everyday ________**  (1) At least a few times a week _________  (2) Only when they feel sick _________  (3) I don’t know ________ |
| B10 | Losing weight usually makes blood pressure | (0) Go up ________  **(1) Go down _________**  (2) Stay the same _______  (3) I don’t know _______ |
| B11 | Eating less salt usually makes blood pressure | (0) Go up ________  **(1) Go down _________**  (2) Stay the same _______  (3) I don’t know _______ |
| B12 | High blood pressure can cause heart attacks | **(0) True _______** (1) False _______  (2) I don’t know ________ |
| B13 | High blood pressure can cause cancer | (0) True _______ **(1) False _______**  (2) I don’t know ________ |
| B14 | High blood pressure can cause kidney problems | **(0) True _______** (1) False _______  (2) I don’t know ________ |
| B15 | High blood pressure can cause diabetes | (0) True _______ **(1) False _______**  (2) I don’t know ________ |
| B16 | High blood pressure can cause a person to have a stroke | **(0) True _______** (1) False _______  (2) I don’t know ________ |
| B17 | Moderate to vigorous exercise 30 minutes/3-5 times a week lowers blood pressure | **(0) True _______**  (1) False _______  (2) I don’t know ________ |
| B18 | Smoking a pack of cigarettes per day will not affect a person’s risk for hypertension | (0) True _______  **(1) False _______**  (2) I don’t know ________ |
| B19 | Motivational interviewing techniques are not useful when guiding a patient to make lifestyle changes | (0) True _______ **(1) False _______**  (2) I don’t know ________ |
| B20 | High blood pressure cannot be cured | **(0) True _______** (1) False _______  (2) I don’t know ________ |
| B21 | A hypertensive individual should strive for a normal blood pressure of 120/80 | **(0) True _______** (1) False _______  (2) I don’t know ________ |
| B22 | A person who has high blood pressure should eat less fat | **(0) True _______** (1) False _______  (2) I don’t know ________ |
| B23 | A person who has high blood pressure should eat more fruits and vegetables | **(0) True _______** (1) False _______  (2) I don’t know ________ |
| B24 | Rate your confidence in detecting and treating hypertension | (0) Very confident _______  (1) Somewhat confident _______  (2) Not confident, I need guidance _______ |

**SECTION 3: Adherence to clinical guidelines and quality hypertension care**

| **Need for and adherence to clinical guidelines** | | |
| --- | --- | --- |
|  | **Statement** | **Choices** |
| C1 | Clinical guidelines are essential for better management of patients with hypertension | 1. Strongly agree 2. Somewhat agree 3. Neutral 4. Somewhat disagree 5. Strongly disagree |
| C2 | I refer to clinical guidelines when I treat patients with hypertension | 1. N/A ( I do not treat patients) 2. Always 3. Very often 4. Sometimes 5. Rarely 6. Never |
| C3a | How confident are you in your adherence to clinical guidelines while taking blood pressure measurements | 1. N/A ( I do not measure Blood pressure) 2. Very confident 3. Somewhat confident 4. Neutral 5. Not very confident 6. Not at all confident |
| C3b | How confident are you in your adherence to clinical guidelines while assigning diagnosis of hypertension? | 1. N/A ( I do not diagnose patients) 2. Very confident 3. Somewhat confident 4. Neutral 5. Not very confident 6. Not at all confident |
| C3c | How confident are you in your adherence to clinical guidelines while educating/counselling hypertensive patients on healthy nutrition/lifestyles/behaviors? | 1. N/A ( I do not privde education/counselling to patients) 2. Very confident 3. Somewhat confident 4. Neutral 5. Not very confident 6. Not at all confident |
| C3d | How confident are you in your adherence to clinical guidelines while prescribing antihypertensive medications? | 1. N/A ( I do not prescribe medications) 2. Very confident 3. Somewhat confident 4. Neutral 5. Not very confident 6. Not at all confident |
| C3e | How confident are you in your overall adherence to clinical guidelines while managing patients with hypertension? | 1. N/A ( I do not treat patients) 2. Very confident 3. Somewhat confident 4. Neutral 5. Not very confident 6. Not at all confident |
| C4a | How confident are you that your colleagues (other providers) adhere to standard guidelines while taking blood pressure measurements? | 1. Very confident 2. Somewhat confident 3. Neutral 4. Not very confident 5. Not at all confident |
| C4b | How confident are you that your colleagues (other providers) adhere to clinical guidelines while diagnosing patients with hypertension? | 1. Very confident 2. Somewhat confident 3. Neutral 4. Not very confident 5. Not at all confident |
| C4c | How confident are you that your colleagues(other providers) adhere to clinical guidelines while educating/counselling hypertensive patients on healthy nutrition/lifestyles/behaviors? | 1. Very confident 2. Somewhat confident 3. Neutral 4. Not very confident 5. Not at all confident |
| C4d | How confident are you that your colleagues (other providers) adhere to clinical guidelines while prescribing antihypertensive medications? | 1. Very confident 2. Somewhat confident 3. Neutral 4. Not very confident 5. Not at all confident |
| C4e | How confident are you that your colleagues (other providers) overall adhere to clinical guidelines while managing patients with hypertension | 1. Very confident 2. Somewhat confident 3. Neutral 4. Not very confident 5. Not at all confident |
| **Quality of hypertension care** | | |
| C5 | I received training on quality improvemvent strategies | (0) True ______ (1) False _______  (2) I don’t know ________ |
| C6a. | We hold regular meetings to review or discuss hypertension performance data of the health facility | (0) True ______ (1) False _______  (2) I don’t know ________ |
| C6b, | If C6a, is True, how often do you hold performance appraisal meetings? | 1. Monthly 2. Quarterly 3. Yearly 4. Other |
| C7 | We utilize data to improve the quality of care to hypertensive patiens. | (0) True ______ (1) False _______  (2) I don’t know ________ |
| C8 | My facility has a system in place to follow up patients with hypertension in their communities | (0) True ______ (1) False _______  (2) I don’t know ________ |
| C9 | My facility has a quality improvement team dedicated to improving the quality of hypertension care | (0) True ______ (1) False _______  (2) I don’t know ________ |
| C10 | We collect hypertensive patients’ feedback to improve their experience of care | 0) True ______ (1) False _______  (2) I don’t know ________ |
| C11 | We use hypertensive patients’ feedback to improve their experience of care | 0) True ______ (1) False _______  (2) I don’t know ________ |
| C12 | At my health facility, healthcare providers collaborate ( use team-based care) while managing patients with hypertension | 1. Strongly agree 2. Somewhat agree 3. Neutral 4. Somewhat disagree 5. Strongly disagree |
| C13 | The facility leadership is committed to improving the quality of hypertension care | 1. Strongly agree 2. Somewhat agree 3. Neutral 4. Somewhat disagree 5. Strongly disagree |

**Answer keys:**

B1 – (1); B2 - (3); B3 – (3); B4-(2); B5-(1); B6- (2); B7 – (0); B8 – (2); B9 – (0); B10 – (1); B11 – (1); B12 - (0); B13 – (1); B14 – (0); B15 – (1); B16 – (0); B17 – (0); B18 – (1); B19 – (1); B20 – (0); B21 – (0); B22 – (0); B23 – (0)
