# Supplementary material for: Exploring patient-, provider-, and health facility-level determinants of blood pressure among patients with hypertension: A multicenter study in Ghana
Source: PLOS Glob Public Health. 2024 Jul 15;4(7):e0002121. doi: 10.1371/journal.pgph.0002121 (PMC11249229; doi:10.1371/journal.pgph.0002121)
Supplement: Attachment — Submitted filename: Rebutal Letter.docx [file pgph.0002121.s005.docx]

| **COMMENTS** | **AUTHOR FEEDBACK** |
| --- | --- |
| **Editorial Comments** |  |
| **Abstract** Line 32: Please add ‘years’ after “…mean (SD) 32 age of 32 (7)…” and …”age of 60.5 (12.7).“  Line 38: Please replace ‘less’ with ‘lower systolic BP’. | Thank you. We made the recommended edits. |
| **Background** Line 48: Please remove ‘chronic’  Line 54: Please improve the syntax: “…ill-equipped with frail health systems that are not prepared…” – remove “ill”; rather replace ‘not prepared’ with ‘unable to’ | Thank you. We made the recommended edits. |
| Line 75: I would suggest that healthcare system (adequate equipment, medication availability, etc.) and healthcare provider (provider inertia, inadequate training, etc.) factors should be considered separately, please. Line 80: “…the facilities for physical exercises, and affordable foods for healthy diets…” I would suggest that these environmental factors are largely influenced by government policy rather than it being the responsibility of the community. Please rephrase.  Similarly, the patient support groups (line 78), while based in the community, would be an extension of the health care system, or has it been established by an independent NGO?  Line 81-82: This is vague: “…worsening status of hypertensive patients.” Please be specific – is it inadequate hypertension control or the development of comorbidities, etc?  Please remove the repetition: Lines 56-62 and Lines 81-97.  Line 98: Please add ‘associated with’ as follows: “…facility-level factors associated with systolic…”  Please tighten, shorten and focus the Background section by clearly articulating the key areas influencing hypertension care (policy and healthcare systems, health care provider and patient factors) and briefly touching on the related variables (medicine shortages, training of staff, etc.). It is currently quite long, somewhat repetitive and disjointed. | We agree that some information was being repeated and the flow was kind of misaligned.  Since we discussed the health system factors in the first and second paragraphs, we have used the opportunity to cut down repetition and abridge the background.  Also, we used the opportunity to separate the healthcare system, and healthcare provider factors.  Further, we made recommended grammatical edits to improve the legibility of the text through-out the background section. |
| **Methods** Please clarify the reason for the convenience sampling design vs. a random sampling technique of the health facilities. | After additional deliberation, our sampling was purposive. I added additional description of our sampling procedure. We could not use random sampling because of the practicality of data collection and lack of resources for collecting data at health facilities located in hard-to-reach areas.  “We purposively selected 25 health facilities at different levels of the health system and with different ownership and from them 15 accepted to join the study.” |
| How were healthcare providers and patients selected? | We added additional details on patient and provider selection and recruitment.  “After arriving at the health facility, a pair of trained enumerators, led by the ranking health facility leader, visited the different health facility units to collect facility-level data about hypertension. We also invited all eligible healthcare providers at their respective health facilities to fill out the online survey but those who were present on the day of data collection were the ones likely to fill out the survey even though the survey was self-administered and online with the possibility of filling it out at home. We encouraged providers to share the survey with their eligible colleagues who were not on duty on our visit to the health facility. Even though we invited clinical care providers from participating health facilities, providers were free to refuse to take the survey.  On the second day, the team recruited patients coming for their usual hypertension care appointments. We worked with the health facilities that signed the MOU of this study to fix the date of data collection on the days when patients are usually scheduled for their hypertension follow-up appointments. The team explained to all patients the purpose of the study and inclusion criteria. We then invited eligible patients to participate. Those who accepted signed the consent forms prior to sitting for survey questions and blood pressure measurements which were part of the data collection. If we were unable to recruit the intended number of patients at a specific health facility, we scheduled an additional visit to the health facility for patient recruitment.” |
| What were the participant (patients and healthcare providers) inclusion and exclusion criteria? | We added additional details to ensure clarity in the inclusion criteria:  “To be included, clinical care providers had to be (1) nurses, physicians, physician assistants, or dietitians; (2) working in hypertension clinics or OPD; and (3) directly involved in managing patients with hypertension. While most nurses were mostly dedicated to the said departments (hypertension clinics or OPD), physicians, physician assistants and dietitians managed patients in other health facility departments as well.  Additionally, from each health facility, we recruited 15 patients with hypertension. To be included, patients had to be (1) have been diagnosed and iniated on any kind of hypertension treatment; (2) receiving ambulatory care at participating health facilities; and (3) be 18 years of age and older. Both male and female patients were eligible to participate in the study. Critically ill or hospitalized patients were not included in the study.” |
| Did all healthcare providers who were interviewed treat patients with hypertension? Did they treat only patients with hypertension at a dedicated hypertension clinic or did they treat all outpatients? | Added more clarity to make this clear and the answer is NO.  “While most nurses were mostly dedicated to the said departments (hypertension clinics or OPD), physicians, physician assistants and dietitians managed patients in other health facility departments as well.” |
| Line 196: Please explain how “bed capacity” contributes directly to hypertension care and BP control? Also, most patients are generally treated at a clinic level. | “The bed capacity variable, though not directly related to outpatient management of patients with hypertension, was utilized as a proxy of the size of the health facility since all facilities included had in-patient capacity. We later evaluated if the size of the health facility was associated with hypertension outcomes.” |
| Line 219: How is “hemoglobinometer or analyzer for hemoglobin testing” related to optimal hypertension care? Please clarify or remove. | These are routine exams conducted for patients with hypertension. We clarified in the text |
| Line 223: It should be Thiazide Diuretics. | Thank you. We made the recommended edit, for this to read “Thiazide diuretics” |
| Analysis: What statistical package was used, please? | We used Stata/BE 17.0 for data analysis. |
| Line 260: How were the “hypertension adherence” variables assessed, please? | Hill Bone high blood pressure therapy scales. We added that specification. |
| Line 281-282: Was the denominator all participants with hypertension? | Correct. The denominator was all patients with hypertension on treatment. |
| **Results**  Line 286-287: ‘Status’ is repeated. Please remove ‘n (%) or mean (SD) (N = 224) n’ from the title and incorporate into the table – Table 1 and other tables. | Thank you. We made the recommended edits. |
| Table 1: Please explain diet, medication and appointment in the footnote. | Thank you. We made the recommended additions. |
| Table 2: What does ‘job orientation’ relate to in the context of hypertension training, please?  Please provide more details on ‘On job training’. | Provided explanation in table 2 and in the explanation text under results |
| Line 290-291: Please clarify if each score was considered adequate, poor or good. | I specified that these scores are high. |
| Lines 304-305 and Tables 3 and 4: Again, please clarify the importance of ‘bed capacity’ in hypertension management and control. | Clarified in the data management section:  “While most nurses were mostly dedicated to the said departments (hypertension clinics or OPD), physicians, physician assistants and dietitians managed patients in other health facility departments as well.” |
| Table 3: Were “Essential antihypertensive medications” all first line therapy, please? | NO. Essential antihypertensive medications include both first- and second-line medications for hypertension. Add this in the table |
| Line 317: Did all “training on cardiovascular diseases” include hypertension management training? | Yes. Added in the text.  “Regarding the health facilities’ hypertension service readiness indicators, 11 (73%) health facilities had at least one healthcare provider who received training on cardiovascular diseases within the last 2 years (this training is expected to have hypertension as one of the topics for the training).” |
| Line 323: Please provide examples of “second-line antihypertensive medication”. |  |
| Lines 322-328: Please discuss 1st line then 2nd line medication; not interchangeably. | Thank you. We made the recommended rearrangement in discussing 1^st^ and 2^nd^ line medications. |
| Line 332-333: Please include the direction of the associations.  Line 338: replace ½ with 0.5 mmHg.   Lines 339-340: Please describe the “diet control scores” – was it related to salt intake specifically/only, etc.?  Line 351: Is the knowledge score considered good or poor? Line 353: “Two-thirds (75%...” – please correct – 75% is three-quarters. Do the authors consider this proportion adequate? | Thank you. We made the recommended edits and added clarifications. |
| **DISCUSSION**  This section is overly long and unfocused. | Thank you. We made the recommended simplified the discussion section to focus on the study findings. |
| After the summary of your findings, please discuss whether this study has adequately identified the variables contributing to hypertension control (good or poor control in this study?) in this population. | We made edits to the introductory paragraph of the discussion and added this concluding sentence:  “Our study found a high rate of uncontrolled BP given that the participants were already on treatment and we identified potentially contributing variables at the patient, provider, and health facility levels.” |
| What are the public health implications of these study findings? What can or should be done to address the challenges and improve hypertension control? | We have listed public health and policy implications throughout the discussion and summarized them in the conclusion.  “In Ghana’s context, interventions targeting the patient-, provider-, and health system levels will be needed to successfully address the hypertension burden. Specifically, in addition to addressing patient level factors that promote adherence to behavioral and medical treatment, the public health experts and policy makers in Ghana should find ways to decentralize hypertension care services, make formal training opportunities available for clinicians who manage patients with hypertension, institute interventions that address the shortage of physician and physician-assistants, and identify and address the root cause of underperformance among private health facilities.” |
| Line 400: Please rephrase: “…factors associated with systolic and…”   Line 402: Please replace ‘exams’ with ‘investigations’.   Line 405: Do you mean education level? | Thank you for the comments. We made the recommended edits. |
| For example, (lines 490-491): is it adequate for a single health care provider to receive hypertension management training seeing that it did not improve hypertension control? As mentioned, the discussion is unfocused; it needs to be targeted to the findings of this study with tangible and practicable recommendations made for improving hypertension care (Lines 478-500). This applies to the entire Discussion section, please. | We simplified the discussion throughout and added this sentences.  “In our findings, many health facilities reported that at least one healthcare provider had received training on hypertension management within two years, yet it is doubtful whether one trained personnel suffices for improved BP outcomes. In our analysis, having at least one healthcare provider who had received training in cardiovascular diseases management in the past 2 years was associated with poor diastolic BP outcomes and in the provider survey, the most common type of training available for healthcare providers was the job orientation for new hires and on the job presentations rather than a structured training for hypertension management. We think that that the poor diastolic BP outcomes among patients at facilities with trained providers could be explained by the new and unexperienced healthcare providers working at those health facilities. “ |
| Lines 522-29: How would these structural changes directly impact hypertension care. Please link your discussion directly/tangibly to hypertension care. | We were explaining why we think private health facilities perform less than public facilities in hypertension management:  “One strategy that has shown to be effective in hypertension management is team-based care—where healthcare professionals collaborate to manage patients with hypertension [54,55]. This could help explain low performance of health facilities where this approach meets implementation challenges.” |
| Line 530-542: Please link cost of care to this study findings. Otherwise, mention as a limitation if it has not been addressed and remove this text. | Thank you for the comment. We added this link to our study:  “Another key aspect that can help explain low performance of private health facilities in Ghana is the cost of care.” |
| Lines 554-556: Has the Service Availability and Readiness Assessment tool been validated to use for hypertension/BP control? | We clarified this: “the Service Availability and Readiness Assessment tool that we utilized has been expert validated in Nigeria [58].” |
| Lines 559-561: Please explain/substantiate how “This study provides data that is vital to entities looking to explore the multi-level factors…” | We have listed public health and policy implications throughout the discussion and summarized them in the conclusion.  “In Ghana’s context, interventions targeting the patient-, provider-, and health system levels will be needed to successfully address the hypertension burden. Specifically, in addition to addressing patient level factors that promote adherence to behavioral and medical treatment, the public health experts and policy makers in Ghana should find ways to decentralize hypertension care services, make formal training opportunities available for clinicians who manage patients with hypertension, institute interventions that address the shortage of physician and physician-assistants, and identify and address the root cause of underperformance among private health facilities.” |
| Line 564: Please explain or rephrase “facility type of management”.  Lines 568-572: I believe that this topic has already been extensively explored in the literature. Please remove/rephrase. Where are the specific research gaps? | We made the edit. And addressed the comment about research gaps in our conclusion section |
| Reviewer 1 |  |
| Congratulations to the authors for such a well planned and executed study of relevance for the management of hypertension  It reads very well and captures the salient findings of what the authors set out to find.  I have no comments than to congratulate the authors on the study. | Thank you very much for taking time to review |
| Reviewer 2 |  |
| This was a very thorough review of issues related to hypertension in Ghana. Some topics that you may also want to expand on is there any additional risk factors for hypertension in Ghana, i.e. hyperlipidemia, diabetes, diet, exercise?  Also are there any rates on the amount of cardiovascular disease/stroke related to untreated hypertension? | Thank you very much for taking time to review. Your questions are very valid and possible next steps and additional angles from which to explore the burden of hypertension in Ghana. |
| If it is possible to expand on what changes could be suggested at the end of the paper, I think that may also be helpful. What can Ghana do to improve rates of hypertension: is it to increase public health clinics?  Given the private clinics do not seem to be having much success, should they be monitored more?  Should there be a decrease number of patients treated per healthcare professional at public clinics?  Should nurses be allowed to do prescribing and have community health workers follow-up on blood pressure checks in patients homes?  Should there be teaching provided in workplaces and schools regarding dietary management and exercise and what blood pressure numbers correspond to hypertension?  In the conclusion, you may want to have some specific steps for how to improve the current rates of diagnosis and treatment of hypertension in Ghana | We have listed public health and policy implications throughout the discussion and summarized them in the conclusion.  “In Ghana’s context, interventions targeting the patient-, provider-, and health system levels will be needed to successfully address the hypertension burden. Specifically, in addition to addressing patient level factors that promote adherence to behavioral and medical treatment, the public health experts and policy makers in Ghana should find ways to decentralize hypertension care services, make formal training opportunities available for clinicians who manage patients with hypertension, institute interventions that address the shortage of physician and physician-assistants, and identify and address the root cause of underperformance among private health facilities.” |
| Reviewer 3 |  |
| While it is known that NCD prevalence is rising at an alarming rate in sub-Saharan Africa, not much is known of the drivers of this and mechanisms that support sufficient NCD management in these settings. Papers that highlight the current state and discuss opportunities for proven effective interventions are surely needed. The authors used a cross-sectional design, purposively sampling health facilities, providers and patients in Ghana to describe determinants of blood pressure among people receiving treatment for hypertension.  They offered no convincing argument to why they sampled purposively (given that hypertension is a common illness) or why they arrived at their sample size.  No report is given of how participants were selected at sites once they met eligibility criteria. The study design precludes us from drawing any conclusions from the data. Despite many statistical tests, they are unable to show any predictors that are both meaningful and statistically significant. | Thank you very much for taking time to review.  We have followed the recommendations from reviewers to provide additional information on our study methods. |
| SPECIFIC COMMENTS:  BACKGROUND: 1. Lines 50-52, are 21% of all patients with hypertension on treatment, or is the denominator here limited to those already aware of their hypertension status?  2. Lines 52-53. It might be helpful to consider framing this around the care cascade – interventions to improve BP control start at preventing increased blood pressure, improving screening and diagnosis and, perhaps most relevant to the context of this paper, improve treatment and control. | Points #1 and 2: We simplified the sentences for clarity:  “In Ghana, approximately 27% of the adult population has hypertension, yet 65% of the patients are unaware of their hypertension status [3]. Among those who are diagnosed (aware) with hypertension, 66% are on treatment and overall, only 6% of all patients with hypertension have a controlled Blood Pressure (BP) [3].” |
| 3. Lines 56-58: While this is true, it lays the blame for complications due to NCDs at the feet of the health system, rather than mention the effects of westernized diet and lifestyle and how corporations lobby against policies and laws that could improve health outcomes at a population level. | Our study explored the factors that play out at different levels (patients, providers, and healthcare system) in hypertension management. The aim of our study was to show that interventions cannot be aimed solely at the patients since health system, and provider- level barriers to care exist. We are confident that we addressed this objective. |
| 4. Line 61: “effective strategies are needed to respond to the rising burden of hypertension”. Agree 100%. However, this creates the expectation that you will suggest such strategies, which by my understanding you did not. | Our study explored the factors that play out at different levels (patients, providers, and healthcare system) in hypertension management. The aim of our study was to show that interventions cannot be aimed solely at the patients since health system, and provider- level barriers to care exist. We are confident that we addressed this objective. |
| 5. Lines 98-100: The aim suggests a paper in 2 parts: 1 describing facility readiness to manage hypertension, the second assessing different factors of having a controlled blood pressure. I would suggest either editing the aim or the rest of the paper so that they match. | We made edits throughout the paper to improve clarity of our methods. We are confident that our study and methods addressed the aims which we ought to address. |
| METHODS 6. Lines 104-106. You cite population growth over the last decade. It may be helpful to readers not familiar with the setting to explain if there is a reason for this growth. Is this at a higher rate than other districts? What are the causes for it? E.g. urbanisation might place people at higher risk of NCDs, and may change how generalisable the findings are. | Thank you for this comment. We reviewed this paragraph to the latest updates and revised the population of 2010 (which we had referenced an incorrect source).  “According to the 2010 population and housing census in Ghana, the Kumasi metropolitan area had a population of 2,035,064 (36% of the Ashanti region’s population) but grew up to approximately 3,768,000 people in 2022 [18,19]. The urban population comprises 61.6% of the Ashanti region population and is growing at the second highest rate in Ghana (After Greater Accra region).” |
| 7. Participants, lines 127-129. Given the very small sample size, I would suggest expanding on how participants at each facility were recruited. Were there any patients who were approached but were not willing to consent? Were they the first 15 patients presenting for the day? The last? Were they sampled randomly from the clinic register? We simply do not know given the information provided in the text. The STROBE Statement you cite earlier in the methods require that you describe the methods of selection of participants. | Here is the description of patients’ recruitment procedure we added:  “We worked with the health facilities that signed the MOU of this study to fix the date of data collection on the days when patients are usually scheduled for their hypertension follow-up appointments. In the morning time, a research assistant explained to all patients (sitting in the waiting area) the purpose of the study and inclusion criteria. We then invited eligible patients to participate, and we enrolled interested patients until we reached the desired number from each health facilitiy. Those who accepted signed the consent forms prior to sitting for survey questions and blood pressure measurements which were part of the data collection. If we were unable to recruit the intended number of patients at a specific health facility, we scheduled an additional visit to the health facility for patient recruitment.” |
| 8. Lines 112-115. Please expand on how you decided on the number of facilities, providers and patients. Did you do a sample size calculation? What variables did you consider and which effect size did you anticipate to be able to pick up given the sample size? | Thank you for this question. Our sample was informed by the Power analysis that is provided under the analysis section.  “The power analysis assumed an Intra-Cluster Correlation (ICC) of 0.1 based on the systolic BP ICC reported by the Phone-based Intervention under Nurse Guidance after Stroke (PINGS) study interim results [28] and a BP control rate of 27% among hypertensive patients on treatment [3]. With these assumptions, and a sample of 254 patients, our mixed-effects logistic regression had 80% power to predict odds ratios of BP control of 1.92 or greater with continuous predictor variables with alpha of 0.05. For dichotomous predictor variables, odds ratios of 3.31, 3.37, and 4.28 were to be detectable if the predictor variable has a 50/50, 40/60, and 20/80 split, respectively.”  While we had assumed the control rate of 27%, our findings of a higher control rate (almost 50%) or half and half reduced our power to detect variables associated with BP control status. We hope the associations with systolic and diastolic BP to be still very informational and essential to inform project implementers. |
| 9. Line 202: Given that the provider to patient ratio is reported as a statistically significant predictor of blood pressure control, if at all possible, I would suggest getting more than 1 months’ estimate of the number of patients seen. These can differ substantially from month to month due to weather, holidays, etc. Having a couple of months and using an average would give a more accurate depiction of the provider patient ratio. | Great idea. We did not collect data on the patient capacity by multiple months. Since we used the same approach across health facilities (which are affected by those weather and holidays the same way), we hope this to be an accurate comparison of them by patients’ BP outcomes across the board. |
| 10. STROBE statement (S1 Table) Apart from social desirability bias, no other biases are mentioned. Given the small sample size and purposive sampling strategy, consideration should be given to how this can introduce bias to your findings. The STROBE statement also requires that you explain how your study size was arrived at. The study size is reported, but how it was decided that it would be 15 participants per facility is not clear. | Responded to this question above.  Also, we added this sentence in the limitations:  Third, the sampling of health facility, provider and patient did not use the ideal random sampling strategy. Despite our attempt to purposively choose a mix of different types of health facilities, the lack of random sampling could have led to us selecting health facilities, providers and patients that are different from the the ones who were not selected to participate or did not choose to participate. These factors could affect the external validity of our findings. |
| RESULTS 11. Table1: I’m not well-versed in this context, but it seems like this population would be different from the general population accessing hypertension care in Ghana. In the studies you cite to compare hypertension control, the mean age is around 40 (Tannor 2020, Tannor 2022, Dosoo 2019), and about 60% of the population is female. No mention is made of these discrepancies and/or how it is addressed. | Thank you for this comment. The studies we compared our findings to included people with and without hypertension, making the age of participants younger than the participants of the current study.  In Tannor 2020 for instance, the mean age of participants with hypertension was 51 and in Tannor 2022, the mean age is 48 years. We have added this age breakdown for readers. |
| 12. Table 3: Here and in multiple other parts of the paper, you refer to antihypertensive medications, but then list classes of drugs. E.g. First line agents that belong the Angiotensin converting enzyme inhibitor class (ACE-I), are usually enalapril or lisinopril. Please list the specific drugs used and not their class. | This is another good strategy one can use. We chose this strategy to standardize the presentation of findings. |
| 13. Line 405-406: No directionality is given – did it lead to improved BP outcomes or worsened BP outcomes? This is also the case in your conclusion. | Thank you for the comment. I made the edits to make the direction clear. |
| 14. In general, the discussion reads as an expanded repetition of the results. I would suggest that you look at the results, highlight some and explain why they are 1) relevant, 2) still valid given the limitations in study design and 3) if they are not generalisable, what can conclusions we reasonably draw from this? | Thank you for the comment. We revised the discussion to be specific to the findings of the study. |
| Reviewer 4 |  |
| This study examined the associations between patient-, provider-, and health facility-level factors and blood pressure among patients with hypertension, and the readiness of health facility readiness for managing hypertension using a cross-sectional design with convenient sampling. The sample size included 15 healthcare facilities, 67 healthcare providers (6 doctors, 6 assistant doctors and 55 nurses) and 224 hypertensive patients who received hypertensive treatment at these facilities.  While I salute the authors for the idea of conducting a study that explore multi-levels of facilitators and barriers in blood pressure management in a low-middle income communities where the rate of blood pressure controlled are considerably low. I have some concerns about this manuscript. | Thank you for taking time to review this manuscript. |
| General:  In my view, the manuscript, especially the Background and Discussion sections is very long, and need to be shorten and focus on what relevant to the study investigation.  Using cross-sectional design with small sample size, and few adjustment variables, the results of regression models should not be interpreted with absolute magnitude effects, but rather present the direction of the effects, especially we don’t know the R2 and the overall significance of the regression model. Details of my comments are below. | We reviewed the background and discussion and shortened them to focus on the aspects of this study.  Further, we added information in the methods section ensure clarity on how we sampled research participants (facility, providers, and patients). |
| Abstract:  Line 35, should be written, ‘…lower systolic BP compared to primary or lower education’. Suggest to remove the word ‘than’  Line 38, suggest to replace the word ‘less’ with ‘lower’  Line 40, remove ‘more’. | Thank you. We made the recommended edits. |
| Background:  What do the authors mean of ‘chronic hypertension’?. I thought hypertension is chronic disease.  Lines 65-68 seems not tie with the study, and can be removed. | Thanks for the comment.  We removed chronic, and removed the mention of AHA/ACC guidelines. |
| Methods:  Reasons why the authors did not  What is the cut-off to define the treatment adhered/complied? Is there any specific reason why the author chose to treat the treatment adherence as a continuous variable and not as categorical variable? | The authors of Hill-Bone tool which we used to measure compliance to high blood pressure dissuade users from categorizing the variable, but rather to treat it as a continuous variable.  “Consistent with the scale developer recommendation, we treated the HB-HBP overall and subscale scores as continuous variables.” |
| Line 168, how did the authors define travel duration to the health facility’? walking duration or transport (public or private) duration? | We considered whole travel duration to the health facility, inclusive of all modes transport, walk alone/ and any type of transportation. We added that in the methods section. |
| Lines 178-184: the authors indicate that they used a 23-item questionnaire developed by their study to determine the knowledge of hypertension management guidelines among healthcare providers.  The authors need to state whether this questionnaire was tested for consistency reliability before using in the main study and provide the questionnaire as supplement material. | We provided the questionnaire as a supplement this time. This questionnaire was not tested for consistency and reliability. |
| Check if lines 237-238 repeated lines 208-210.  In Data analysis session, the authors stated that ‘duration with hypertension diagnosis’ was included as an exposure of interest, if I am not mistaken, but in the Tables of Results, the authors reported treated it as adjustment variable, and this needs to be explained. | One asks the health facility if at least one provider had received CVD training in the last two years, and another variable is provider specific. The later, asks the specific provider if they had received CVD management training and the type of training they received. So, this is not a repetition.  Thank you for helping catch an oversight in the reporting of variables. We included duration with hypertension diagnosis in the variables of interest rather than a covariate. |
| Results:  Again, I disagree with the way the authors interpreted the results. There would be bias by sayings ‘Having a secondary level of education was associated with roughly 8 mmHg lower systolic BP’. Rather simply say that ‘Having a secondary level of education was associated with lower systolic BP’. SD is always positive, so please remove (+) symbol in front of the values. | Thank you for the comment, however, despite any limitations of the study, we chose to interpret our findings as we found them (abiding on the statistical rules of interpreting coefficients of a linear regression analysis).  We have corrected (removed) the + and ± before SD. |
| Lines 346-347, the author indicate that ‘in the unadjusted and fully adjusted mixed-effects logistic regression models, only the duration of travel to the health facility remained significant.’ This is incorrect as Table 1 showed no difference in terms of travel duration to health facility between patients with controlled BP and uncontrolled BP (p=0.125) if I am not mistaken.  In fact, there is no need to fit regression models for BP control because the bivariate analysis showed that no difference between patients with controlled BP and uncontrolled BP for risk factors investigated. The different direction effects of travel duration with blood pressure control maybe due to chance. | Great suggestion.  We did not use Forward Elimination Stepwise Analysis in the selection of variables to include in the full model. Rather, we included all variables pertinent to our working conceptual model of variables associated with blood pressure levels and BP control. We then conducted model checking to ensure that the models fit the data correctly. |
| Table 6, ‘Facility size by bed count’, the third level is supposed to be ≥100 bed. | Thank you for this edit recommendation. We made corrections. |
| **Discussion:**  In my view, this section appears not very focused, for instance, lines 426-439; lines 494-496; 498-500 are not related to the study. | Thank you for the comment. We revised the discussion to be specific to the findings of the study. |
| The third paragraph, the authors discussed the relationship – education-economic status and blood pressure. But in this study, the authors found education and not employment was associated with blood pressure, and this needs to be discussed. | Thank you for the comment. We revised the discussion to be specific to the findings of the study. |
| Line 464: ‘These findings corroborate the previous literature.’ and the author referred to a study by Sarfo (2020) who found adherence to treatment was associated with higher odds of BP control. Isn’t it contradict with this study where the authors found no association between treatment adherence scores and BP control. | Adjusted the paragraph to align with our findings. As we stated in the introduction, while the BP control is preferred, any reduction in BP is preferred than no reduction. Our findings are reflective of this statement. |
| Line 469, why does smoking habit hinder hypertensive treatment adherence? | While we mention this as one of the patient-level factors of BP outcomes that was reported in other studies, our data did not allow us to explore it. We hence did not focus on explaining the process by association between poor adherence and smoking habit. Further, this is association and not necessarily a causal pathway in either direction. |
| The suggestion of the implementation of task-shifting where training nurse to manage hypertension seem odd with the finding that there was no association between patient-nurse ratio and BP outcome. | Great question. Task-shifting is more than giving tasks to nurses. It includes providing them with ongoing and continuous training (something which was sub-optimal in the current study). This could explain the lack of association for the variable patient-to-nurse ratio. |
| Another important limitation of this study is that study was conducted in a single district with convenient sampling, and thus the results are not generalisable to entire province or entire Ghana. | Thank you for the comment. We addressed the concern of generalizability in our study limitations.  “Third, the sampling of health facility, provider, and patient did not use the ideal random sampling strategy. Despite our attempt to purposively choose a mix of different types of health facilities, the lack of random sampling could have led to us selecting health facilities, providers, and patients that are different from the the ones who were not selected to participate or did not choose to participate. These factors could affect the external validity of our findings.” |
