# Supplementary material for: Exploring patient-, provider-, and health facility-level determinants of blood pressure among patients with hypertension: A multicenter study in Ghana
Source: PLOS Glob Public Health. 2024 Jul 15;4(7):e0002121. doi: 10.1371/journal.pgph.0002121 (PMC11249229; doi:10.1371/journal.pgph.0002121)
Supplement: Attachment — Submitted filename: Response to Reviewers.docx [file pgph.0002121.s006.docx]

**Response to reviewers**

**Journal Requirements:**

1. Please amend your detailed Financial Disclosure statement. This is published with the article. It must therefore be completed in full sentences and contain the exact wording you wish to be published.

We made edits to the funding statement for further clarity.

a. State what role the funders took in the study. If the funders had no role in your study, please state: “The funders had no role in study design, data collection and analysis, decision to publish, or preparation of the manuscript.”

Added.

b. If any authors received a salary from any of your funders, please state which authors and which funders.

If you did not receive any funding for this study, please simply state: “The authors received no specific funding for this work.”

Additional Editor Comments (if provided):

Line 49: ‘…leading causes of death, cardiovascular disease, and stroke’. Please explain clearly Is CVD not the cause of death being referred to and does stroke not fall under CVD? This is the impression created by this text.

We made the edit which we hope improved clarity.

Lines 56-58: Please improve the sentence construction.
We made the edits.

Line 59 What ‘public health system initiatives’ are currently in place? Or are you referring to generic efforts?

For brevity, we took it out since it is not necessary to convey the message about low population awareness of hypertension.

Line 71-72: Improve syntax of the entire sentence e.g., ‘…beneficial to explore factors not only affecting hypertension control…’

Improved

Lines 75-77: What about high caloric intake and obesity?

Added

Line 90: replace ‘grew up’ with ‘increased’.

Replaced

Line 162: Please include the HB-HBP scale as a supplementary table in this paper.

Included

Lines 185-186: Please include the ‘23-item questionnaire’ as a supplementary table as well.

Already part of supplementary documents. S2 Table.

Line 228: Correct spelling of ‘routinely’.

Corrected.

Table 1:
Improve syntax – it should read ‘duration of hypertension diagnoses’.
p-value should be written and reported as <0.001 and not 0.000; please correct.

Corrected.

Line 340: replace ‘exams’ with tests.

Replaced

Table 5:
‘Length of diagnosis’ – include the condition.
Please amend to ‘Compliance with high blood pressure therapy’.

In response to feedback from Reviewer #3, we have changed the formatting and number of tables. We have however, throughout, made the recommended edits to the text referenced above.

Line 370: In line with how you’ve presented the data, amend the text ‘…were two times less likely…’ to ‘were half as likely to…’

Edited

Line 376: remove ‘which is impressive’ – this is best discussed in the Discussion section with reasons provided as to why the authors consider this ‘impressive’.
This does not correspond with the confidence in diagnosing and treating hypertension. Please comment in the Discussion.

Removed.

Line 412: replace ‘oddly’ with ‘unexpectedly’. There are likely various reasons for this finding which does not make it ‘odd’.

Replaced

Line 425: include ‘one’ BP measurement equipment. Note, that a single working BP machine is sub-optimal.

In compliance to the recommendations of reviewer #3, we made additional analysis and presented additional findings about medications and equipment. In Table 3 (Line 339), We added that the median number of BP devices was 2 (Which is, admittedly, still not ideal, in addition to the fact that the availability of large and small-size cuffs was minimal) – we added these data in Table 3.

Line 439: replace ‘this’ with ‘latter study’ to prevent confusion.

Corrected.

Lines 437-449: briefly summarise hypertension control in other studies rather than listing each one individually.
Done

Lines 450-455: The flow is poor and confusing – lower SBP in your is followed by a study from Accra and back to your study with no significant associations found. Please improve for clarity.
For brevity and focus of the results and discussion section, we removed the discussion about the association between education and BP outcomes. Our initial goal was to use education as a covariate not an exposure of interest. We hence removed it from the results table, results section, and the discussion.

Line 455-464. This text is rambling and without a clear message. Please summarise succinctly your message about education, wealth and BP control with direct comparison and relevance to your study findings. Please shorten.

We removed the section since it was not in line with our initial plan of analysis. Education was planned to be used as a covariate to use for adjustment in the full models.

Lines 466-489: This paragraph is a page long, rambling and unfocused. It discusses distance travelled to clinics, strengthening of healthcare services, waiting times, etc. as well as an example from Rwanda where removing distance barriers made no impact on BP control, without discussing this contrasting finding. It should be one paragraph, one idea. What is the key message in this paragraph? Please summarise clearly and succinctly. Please shorten.

We shortened the paragraph to 12 lines down from 24 lines

Lines 489 onwards: To avoid repeating myself, I suggest that the authors review the rest of their Discussion in line with the recommendations above. Note, this suggestion was made in the previous review as well but has not been adequately addressed. I suggest that an experienced science writer/researcher review the text before resubmitting again, please.

We appreciate your recommendation and a person experienced in science writing re-reviewed the paper. Further, we sent the manuscript to a professional editor to ensure clarity of English language.

**COMMENTS TO THE AUTHOR**

**Reviewer #2:**

Thank you for the revision and great overview of the need to address HTN in LMICs. A few thoughts on this, beta blockers as listed are not considered as best first line antihypertensive treatment for hypertension unless there was combination therapy or specific indications.

We have reviewed the literature and removed beta blockers from the list of first line antihypertensive medications and re-run the analysis.

Also, in regard to the affluent having increased BP, is there a correlation to their outcomes in regard to STEMIs, CV disease overall? In regards to outcomes as they have been written, what cardiovascular outcomes you referring to? Stroke and MI?

Considering other reviewers’ critiques of the section and it being unfocused, we have removed it.

Also, for the conclusion, may want to address the need for specific partnerships to address HTN and incorporate it into SDG goals as well (noncommunicable diseases).

Good comment. Since we did not raise it in the discussion (to cut down on the word count) we did not introduce it in the conclusion, but this is very true. Addressing hypertension could help reach SDG3.

**Reviewer #3:**

1. Despite concerns voiced by multiple reviewers in the previous round of review, the main statistical analysis has remained unchanged as far as I can discern. No crude estimates are provided in the main paper and it is not clear how the authors determined which variables to include in the adjusted analysis. The authors also draw inappropriate conclusions from the regression, given limitations in study methodology and overstate their findings. While they have included how they completed their sample size calculation in this version, they still do not cite or provide a reason why they used the expected effect sizes they did (lines 281-288). This is especially relevant as these differ from what they found in their study.

We initially had an oversight in our write up of the power analysis. We have corrected the typo. Our initial power analysis considered the BP control rate among patients on treatment of 45.5% rather than 27%.

Also, we provided explanation (Lines 268-283) of how we selected variables to include in the model. Guided by the WHO health systems framework, we selected available variables representative of building blocks of the health systems (medications, patient-to-provider ratio, travel duration, availability of hypertension clinic...). Other variables we wanted to include (re: equipment) rendered the model unstable and we omitted them, as we explain in the analysis section.

We did not use a particular p-value cut-off to consider the variables to include in the final model.

We have re-formatted the tables, added crude estimates, removed some covariates which were not key exposures, and left the main predictors as planned in the analysis section.

2. Saying that increased provider training is bad for patient outcomes is a big statement. I do not think the study methodology and results support it. Given the number of variables the authors included in their analysis, they were likely to find something that is statistically relevant. That does not in and of itself make it true or meaningful. The variable itself is problematic – if most of the training is from onboarding, providers with the least experience fall into this category. I therefore do not think it is an adequate measure of knowledge or experience and use of it introduces bias. While the authors mention this briefly in their discussion, I don’t think enough justification is provided to include it.

Considering your feedback on the interpretation of provider training, we have removed it from the current study due to its potential to bias our interpretation of findings. Thank you for taking time to review and comment.

3. Multiple reviewers raised concerns on the length and focus of the discussion. It has been edited in some parts, but it still spans 10 pages and still lacks focus.

We have cut the discussion further down to 5 pages and made edits to make it focused

4. While it is the authors’ prerogative to decide how they group drugs provided in the management of hypertension, what they are listing are not antihypertensive medications as mentioned, but classes of drugs. To be using the correct terminology, they either need to refer to them as classes of drugs, or list the antihypertensives separately.

Thank you for your comment. We have added a Table listing the specific medications that were available at health facilities. Please check table 4.

**Reviewer #4:**

The authors have fully addressed my comments. I am happy with the revised version, although some minor revision needed mainly in terms of the language, but I will leave this to the Editor.

Thank you.
