# Supplementary material for: Exploring patient-, provider-, and health facility-level determinants of blood pressure among patients with hypertension: A multicenter study in Ghana
Source: PLOS Glob Public Health. 2024 Jul 15;4(7):e0002121. doi: 10.1371/journal.pgph.0002121 (PMC11249229; doi:10.1371/journal.pgph.0002121)
Supplement: Attachment — Submitted filename: Revisions point by point.docx [file pgph.0002121.s007.docx]

**Additional Editor Comments (if provided):**

Overall:
Please write all text in the past tense, and not the present tense.
Please check syntax throughout the paper.
Please streamline the paper further.

Line 53: ‘the leading causes of death’ – what does this standalone phrase refer to, please? Improve sentence clarity.

Edited

Lines 515-516: Did these hospitals treat all patients with hypertension or those mainly with complicated hypertension. If the latter, then it is not comparable to hypertension control in other settings. Were these patients recruited from routine hypertension clinics?

While in other places such as the US, private health facilities care for patients with complicated conditions than government health facilities, it tends to be a flip in low-income countries such as Ghana. In Ghana, private health facilities often do not have resources to manage complicated or urgent cases of hypertension (these will mostly be found at government health facilities). Cardiologists and other specialists often work part time at private health facilities and full-time at government health facilities. Patients usually go to private health facilities hoping to be seen by a specialist with relatively shorter waiting time than in government health facilities which often longer. Health-wise, patients who seek care at private health facilities will not be different from people who seek care in government health facilities. In this study, we found that 38% of patients had or had at least one of the end-organ damage hypertension complication (kidney disease- 0.5%, stroke – 6.2%, vision problems – 18%, cardiac disease – 11%, neuropathy-8%).^1^ Among patients at government hospitals this was roughly 50%, private-35% and CHAG – 26% - again highlighting that people with complicated hypertension are not necessarily at private health facilities.

1. Byiringiro S, Hinneh T, Sarfo FS, Commodore-Mensah Y, Dennison Himmelfarb CR. Abstract 15917: Burden of End Organ Damage Among Patients With Hypertension and Associated Patient- and Facility-Level Factors in Ghana. *Circulation.* 2022/11/08 2022;146(Suppl_1):A15917-A15917.

Lines 562-567: Divide into 2 sentences and improve syntax.

Done. Thank you for this recommended edit

Line 588: Please remove ‘the’.

Removed

Line 592: Why are this study findings different from the literature?

We cannot find which part of the manuscript this comment is addressing, since there is no line 592 in the text part of the manuscript submitted.

Line 622-624: Improve grammar: ‘results into a rush in consultation…’

We found it and edited this part.

Line 630: Improve syntax.

We cannot find which part of the manuscript this comment is addressing, since there is no line 630 in the text part of the manuscript submitted.

[Note: HTML markup is below. Please do not edit.]

Reviewers' comments:

Reviewer's Responses to Questions

**Comments to the Author**

1. If the authors have adequately addressed your comments raised in a previous round of review and you feel that this manuscript is now acceptable for publication, you may indicate that here to bypass the “Comments to the Author” section, enter your conflict of interest statement in the “Confidential to Editor” section, and submit your "Accept" recommendation.

Reviewer #2: All comments have been addressed

Reviewer #3: (No Response)

2. Does this manuscript meet PLOS Global Public Health’s [publication criteria](https://journals.plos.org/globalpublichealth/s/criteria-for-publication)? Is the manuscript technically sound, and do the data support the conclusions? The manuscript must describe methodologically and ethically rigorous research with conclusions that are appropriately drawn based on the data presented.

Reviewer #2: Yes

Reviewer #3: No

3. Has the statistical analysis been performed appropriately and rigorously?

Reviewer #2: Yes

Reviewer #3: No

4. Have the authors made all data underlying the findings in their manuscript fully available (please refer to the Data Availability Statement at the start of the manuscript PDF file)?

The [PLOS Data policy](https://journals.plos.org/globalpublichealth/s/data-availability) requires authors to make all data underlying the findings described in their manuscript fully available without restriction, with rare exception. The data should be provided as part of the manuscript or its supporting information, or deposited to a public repository. For example, in addition to summary statistics, the data points behind means, medians and variance measures should be available. If there are restrictions on publicly sharing data—e.g. participant privacy or use of data from a third party—those must be specified.

Reviewer #2: Yes

Reviewer #3: Yes

5. Is the manuscript presented in an intelligible fashion and written in standard English?

PLOS Global Public Health does not copyedit accepted manuscripts, so the language in submitted articles must be clear, correct, and unambiguous. Any typographical or grammatical errors should be corrected at revision, so please note any specific errors here.

Reviewer #2: Yes

Reviewer #3: Yes

6. Review Comments to the Author

Please use the space provided to explain your answers to the questions above. You may also include additional comments for the author, including concerns about dual publication, research ethics, or publication ethics. (Please upload your review as an attachment if it exceeds 20,000 characters)

Reviewer #2: (No Response)

Reviewer #3: GENERAL COMMENTS
Thank you for the edits to this manuscript. I want to reiterate that papers that highlight the current state of hypertension management and discuss opportunities for proven effective interventions are surely needed. I still think that describing what you found at each facility and provider knowledge would be a strong paper in and of itself. Most of the remaining concerns are related to the patient-level data.

Thank you for this comment. We are hoping to address your concerns in the current submission.

SPECIFIC COMMENTS:
1. Line 41: This does not fit with the message provided in the discussion.

It is not exactly clear what on Line 41 does not align with the discussion. We hope that we addressed this through the additional revisions we conducted.

2. Line 51-54: There’s jumping around between what the denominator of the reported percentage is, which makes it hard to follow. Looking at this, you’re saying only 65% are aware of their hypertension status, but 66% are on treatment. How is that possible? I suggest editing for consistency and clarity.\

We have reworded it to this:

In Ghana, approximately 27% of the adult population has hypertension [3]. Additionally, there is a low prevalence of hypertension awareness, diagnosis, and treatment which is one of the reasons why only 6% of all people with hypertension have controlled BP [3].

3. Line 55: Suggest using term “resource constrained” rather than “frail” when referring to health systems.

Edit made.

4. Line 160: Has this tool been validated in this setting?

This tool has not been validated in this setting but has been utilized widely in this setting.

5. Lines 191-198: Have you considered the impact around the “know-do” gap? It is well described in the literature that even when providers know what to do, that it doesn’t necessarily translate into practice.

The questions about attitude towards the guidelines purported to address the “know-do” gap

6. Lines 230-231: In the response to reviewers, you stated that you’ve listed these, but here you still refer to classes of drugs as first line medications.

We removed the classes from the manuscript and refer to the first line drugs by their names.

7. Lines 235-237: Shouldn’t education be ordinal, given that it does fall into categories that can be ordered?

Thank you for your comment. We understood that ordinal and nominal variables are both categorical variables. We have renamed it as “ordinal” to be more specific as recommended.

8. Line 247: Is it therefore fair to assume that all these variables followed a normal distribution? If not, please consider reporting medians ad IQR

Thank you for the comment. We reported medians and IQR for non-normally distributed data. We added this in the description of analysis.

9. Line 250: Again, reporting of mean implies normal distribution. Were the scores of knowledge normally distributed?

We made corrections for clarity.

10. Lines 281-288: I would suggest that the power calculation/sample size, since it would have been done prior to participant selection, be moved to line 102.

We moved it.

11. Table 1: Usually, p-values are not reported in the baseline characteristics table.

We removed them.

12. Table 1: According to the creators of the scale, “The Hill-Bone Compliance to High Blood Pressure Therapy Scale is one of two Hill-Bone Scales. This is the original, 14-item Hill-Bone Scale developed to assess patient behaviors for three important behavioral domains of high blood pressure treatment (the three sub-scales of the original scale): Appointment Keeping (3-items), Diet (2-items), Medication Adherence (9-items).” However, you report 2 questions for appointment keeping and 3 for Diet? Can you please clarify?

Thank you for the comment. We had a similar challenge before initiating the study. Here is the correspondence we relayed to the tool developer. We followed the subscales that are on the tool itself rather than what is on the website (which is what the developer recommended). Appointment Keeping (2-items), Diet (3-items), Medication Adherence (9-items) (find more details in the image below).

13. Table 3: Technically, the urine dipstick for protein, glucose and ketones would be a single test strip. Is this not the case in Ghana?

Often, but not always, especially in LMICs where health facilities tend to receive the supplies from different vendors from time to time.

14. Table 3 Notes: Here you are still listing classes of drug when it should be the medications.

Changed

15. Line 361-362: Does high BP therapy refer to antihypertensives? I would suggest reconsidering the phrasing here and to refrain from using “high BP therapy” as a technical term.

Compliance with high BP therapy scores, refers to Hill-bone compliance to high BP therapy (HB-HBP) scores. High BP therapy in our context is more than medications. In addition to medications, therapy as captured by HB-HBP includes salt intake reduction.

16. Table 5: Again, here the number of questions for different sections of the compliance score are not in line with literature describing number of questions per section.

We provided description to this issue above.

17. Lines 376-378: CI still wide and only just significant. what happens if you stratify by urban vs rural?

Thank you for this question. As we noted in the “setting” subsection of the methods, “We conducted this study in the Kumasi metropolitan area which is mostly urban and houses the capital city of the Ashanti region in Ghana.” We hence cannot stratify by rural vs urban.

18. Lines 381-382: I still don’t understand how the authors have made the determination that the mean score was “relatively good”. Relative to what? Having looked at the questions provided, they seem basic and having providers score <80% seems worrying.

We removed the “relatively good” and left it to the discussion section. We agree that less than 80% is warring among clinicians.

19. Line 415-420: Please confirm that the values reported in text and tables match. Please also review the abstract for this.

The values in the text match the values in the tables.

20. Line 450-453: Please add all the numbers used in this calculation.

We made edits to this section.

“For additional context, only 30% of patients aware of their hypertension status were initiated on treatment [3]. Since only 35% of people with hypertension are diagnosed, the overall estimate of patients with controlled BP narrows down to 6%, further demonstrating a wide gap in hypertension management [3].”

21. S3 Table: Please indicate which response you considered to be correct.
Added. Thank you

RESPONSES TO REVIEWERS:
22. In response to question 1 to reviewer 3, this still does not provide the information on WHY the estimate in your power calculation and what you found in your study differ so much. Which one do you think is correct? Why do they differ so much? I was not able to find this information in the methods, response or discussion.

In our power calculation, the BP control among patients on treatment was 45.5%. In the current study, we found 49% had controlled BP which is 3.5% points higher. In the discussion, we discussed that according to previous literature “BP control among patients on hypertension ranges between 23% and 49%”. Our study was right in this range.

Our explanation was this (lines 534-536) in the discussion section: “It is possible that studies recruiting patients in healthcare settings may report lower rates of BP control because people tend to seek care when they feel sick—possibly from complications of uncontrolled BP.”

23. While adding table 4 is useful, this does not address the underlying concern that the terminology used is not correct. You would have to refer to that list every time you mention anti-hypertensive medications. I’ve made note of instances where this is still problematic above.

This time, we are hoping that we addressed your concern with terminology.
