# Supplementary material for: Exploring patient-, provider-, and health facility-level determinants of blood pressure among patients with hypertension: A multicenter study in Ghana
Source: PLOS Glob Public Health. 2024 Jul 15;4(7):e0002121. doi: 10.1371/journal.pgph.0002121 (PMC11249229; doi:10.1371/journal.pgph.0002121)
Supplement: Attachment — Submitted filename: Rebutal letter.docx [file pgph.0002121.s008.docx]

**Point by point feedback to reviewer comments**

Reviewer #3: Thank you for your responses to my previous comments. I have no new ones.

Thank you for taking time to review

Reviewer #4: In this revision, I have only comments regarding the presentation of Table 1.
Since the author present two subgroups of uncontrolled and controlled hypertension, it makes sense to include the column of P-values comparing the characteristics of these two.
However, with non-parametric data which the authors presented in median (IQR), the use of t-test and ANOVA test are not appropriate, and Kruskal Wallis test should be used.

Thank you for this important feedback. We provided the p-values in Table 1.

Also, we evaluated our analyses and made sure to re-run the tests using Kruskal Wallis test for continuous variables that are not normally distributed. While there were no new associations identified, we adjusted our description of analysis method in the analysis section.
